# Transformational Leadership Style, Structural Empowerment and Staff Outcomes in Nursing Home Settings

**DOI:** 10.1002/nop2.70646

**Published:** 2026-06-22

**Authors:** Milja Niinihuhta, Anja Terkamo‐Moisio, Tarja Kvist, Arja Häggman‐Laitila

**Affiliations:** ^1^ Department of Nursing Science University of Eastern Finland Kuopio Finland; ^2^ University of Helsinki and Helsinki University Hospital Helsinki Uusimaa Finland; ^3^ Department of Public Health University of Helsinki Helsinki Uusimaa Finland

**Keywords:** leadership, nurse leader, nursing home, nursing staff outcomes, older people care, structural empowerment, transformational leadership style, work‐related well‐being

## Abstract

**Aim:**

This study aimed to describe transformational leadership style, structural empowerment, and staff outcomes and associated factors. A further aim was to explore factors that potentially explain commitment to the workplace and the profession in the context of nursing homes.

**Design:**

An explorative cross‐sectional study design with two datasets.

**Methods:**

The target group consisted of staff working in 24‐h, long‐ or short‐term care in Finnish nursing homes. Data were collected between November 2022 and April 2023 through a survey, including background questions and two instruments: The Transformational Leadership Scale and the Conditions for Work Effectiveness Questionnaire II for structural empowerment. The data were analysed by descriptive and multivariate statistical methods.

**Results:**

A total of 97 nurse leaders (response rate 76%) and 503 care personnel (response rate 21%) participated. Transformational leadership, work‐related well‐being, overall health, and job satisfaction were rated as good. Nurse leaders reported higher scores than care personnel. Nurse leaders assessed their structural empowerment as moderate. They rated opportunity and information as the strongest dimensions of structural empowerment, while resources were the weakest dimension. Care personnel reported a lack of opportunities to participate in decision‐making and to advance their careers. For nurse leaders, factors explaining commitment to their current workplace were work‐related well‐being and commitment to the profession, while for care personnel these were valuing one's own work, nurse leaders' transformational leadership, work‐related well‐being, and commitment to the profession. The factors explaining care personnel's commitment to the profession were work‐related well‐being, valuing one's own work, and commitment to the current workplace.

**Conclusion:**

Transformational leadership and structural empowerment appear to be important factors associated with positive staff outcomes in nursing home settings. Nursing homes are complex and currently understudied environments, highlighting the need for further research to explore different associations and potential explanatory factors in this context.

## 1 Introduction

Nursing staff constitute the largest professional group in healthcare (Friganović et al. 2019; Seabra et al. 2019). Current and future challenges include a shortage of nurses, turnover, and the low attractiveness of the profession. For example, according to the World Health Organization (2020), by 2030 there will be a shortage of approximately 6 million nurses worldwide. The reasons for the shortage include the expectation that one in six nurses will retire (WHO 2020), growing demand for nurses due to population growth and ageing (WHO 2020; Xu et al. 2023) and a consequent reduction in the number of people applying to become nurses (Marufu et al. 2021). The drivers of shortages, turnover, and low attractiveness of the profession are similar in nursing around the world, regardless of cultural differences. These include problems with staffing, leadership, support, and recognition. (Marufu et al. 2021). High workloads, lack of career progression and educational opportunities, poor work‐related well‐being, and low job satisfaction are associated with nursing staff turnover (Marufu et al. 2021; Xu et al. 2023).

Leadership has been identified as a critical factor in positive staff outcomes such as commitment, retention in the profession, work‐related well‐being, and job satisfaction (Mabona et al. 2022). Relational leadership styles, such as transformational leadership style and their associations with staff outcomes are most widely studied (Hult et al. 2023). Studies have consistently shown that transformational leadership style is associated with decreased turnover intentions (Alilyyani et al. 2022; Cummings et al. 2018), increased workplace safety climate (Farag et al. 2017), and work‐related well‐being and job satisfaction (Cummings et al. 2018; Hult et al. 2023). Transformational leaders possess good communication skills (Lee et al. 2023), and emotional intelligence (Frangieh and Jones 2022) and are willing to mentor and teach nurses (Kvist et al. 2019). They are visionary, supportive, show interest in clinical work, and encourage nurses' autonomy, decision‐making and contribution to patient care and teamwork. They foster trust in both the organization and the leader. (Cummings et al. 2018; Dall'Ora et al. 2020; Hult et al. 2023).

Nurse leaders can also influence positive staff outcomes by creating a work environment that enhances staff's structural empowerment (Spence Laschinger et al. 2014). Previous studies indicate that structural empowerment is associated with strengthened work‐related well‐being among nurses (Valle et al. 2021) and reduced stress (Al Sabei et al. 2022; Jafari et al. 2021), emotional exhaustion (Jafari et al. 2021), and mental health symptoms (Saleh et al. 2022). Structural empowerment is also associated with diminished intention to leave (Valle et al. 2021) and increased job satisfaction (Abel and Hand 2018; De Almeida and Orgambídez 2019).

Structural empowerment involves access to information, support, resources, and opportunities for growth and professional development in the workplace. Structural empowerment enables employees to contribute to achieving organizational goals (Abel and Hand 2018; De Almeida and Orgambídez 2019). Greater access to, for example, resources and support can enhance nurses' job satisfaction and allow working with greater autonomy (De Almeida and Orgambídez 2019). Access to opportunities for professional growth means, for example, opportunities for continuous education, and access to resources refers, for example, to the availability of financial means. Access to information indicates having both formal and informal knowledge in the organization, and access to support includes feedback and guidance from superiors or peers. Formal power results from specific job characteristics, such as flexibility or adaptability, and informal power results from, for example, social connections or development of communication channels within the organization. (Laschinger 2012).

Work‐related well‐being has physical, psychological, social, and emotional aspects, and is part of the comprehensive well‐being of humans. It includes aspects both inside and outside of the workplace and may vary, for example between disciplines or organizations. (Buffet et al. 2013). Work‐related well‐being among nurses is often studied in terms of ill‐being, for instance through the lens of burnout, stress, depression, and anxiety (Jarden et al. 2020; Patrician et al. 2022). Nursing is a demanding profession, and nursing staff are at risk of mental health problems such as burnout and stress. Stress and burnout levels among nurses are moderate to high (Grabbe et al. 2020; Khatatbeh et al. 2022), while nurse leaders experience moderate stress levels (Labrague et al. 2018) and moderate to high levels of burnout (Kelly et al. 2019). Problems with work‐related well‐being and high levels of sickness absence decrease effectiveness and quality of care in the health care sector and increase costs (Grabbe et al. 2020; Hult et al. 2023). For example, a study in nursing home settings (Chao 2019) found an association between high levels of depression among nurses, low levels of client satisfaction and perceived quality of life, and significant depressive symptoms among older people.

It is important to identify the essential aspects that nurse leaders should focus their attention on in complex work environments to ensure positive staff outcomes as these are in turn connected to positive patient outcomes (Khatatbeh et al. 2022; Marufu et al. 2021). Previous research on them has often focused on acute contexts in health care (Jarden et al. 2020; Khatatbeh et al. 2022; Marufu et al. 2021; Niinihuhta and Häggman‐Laitila 2022), paying less attention to nursing home settings even though leaders in this field are struggling even more with the shortage of nursing staff than are their colleagues in more acute care contexts (Gibson and Greene 2020). This study addresses a gap in the research examining the relationships between nurse leaders' structural empowerment, transformational leadership style, and staff outcomes, namely commitment, work‐related well‐being, overall health, job satisfaction, and valuing one's own work, in the context of nursing home settings.

## 2 The Study

### 2.1 Aim and Objective

This study aimed to describe transformational leadership style, structural empowerment, and staff outcomes and associated factors. A further aim was to explore factors that potentially explain commitment in the workplace and profession in the context of nursing homes.

The research questions were:

1. How do the staff evaluate the transformational leadership style, structural empowerment, and staff outcomes?
2. How are the sociodemographic factors and setting characteristics associated with staff's evaluations?
3. How are nurse leaders' transformational leadership style and their structural empowerment associated with staff outcomes?
4. Which factors explain staff's commitment to the current workplace and profession?

## 3 Methods

### 3.1 Design

An explorative cross‐sectional study design with two data sets was employed and reported following the Strengthening the Reporting of Cross‐sectional studies in Epidemiology (STROBE) checklist.

### 3.2 Research Context and Target Group

The target group for this study was nursing staff working in 24‐h, long‐ or short‐term nursing home settings in a large organization in Finland, referred to as nursing homes in this study. In Finland, local authorities must organize long‐term care for older persons primarily in the person's private home or other home‐like place of residence. Long‐term care can be provided in the form of institutional care only if there are medical grounds or if it is otherwise justified. (Act on Supporting the Functional Capacity of the Older Population and on Social and Health Care Services for Older Persons 980/2012 § 14a) (2012). Long‐term nursing home settings provide both short‐ and long‐term 24‐h care, as defined in the Social Welfare Act 1310/2014 (Finnish Institute for Health and Welfare 2024). In 2023 the largest professional group in immediate patient care in long‐term nursing home settings in Finland were practical nurses (69%), followed by care assistants (10%), other professions (8%), such as physiotherapists, occupational therapists, or social advisers, and registered nurses (7%) (Finnish Institute for Health and Welfare 2024). Practical nurse is an upper secondary education with a duration of 2.5 years (180 ECVET competence points), and achieving basic qualification for care assistants takes approximately 1 year (60 ECVET competence points). In comparison, duration for Bachelor of Nursing or Bachelor of Health Care (physiotherapists and occupational therapists), Bachelor of Social Services (social advisor), and Bachelor of Social Services and Health Care (geronoms, older people care professionals in geriatric social work) is 3.5 years (210 ECTS credits) (Finnish National Agency for Education 2025).

This study included two data sets of staff, nurse leaders (*N* = 129) such as nurse directors, nurse managers, deputy nurse managers, nurse team leaders, as well as immediate care personnel (*N* = 2492) such as registered and practical nurses, physiotherapists, social advisors, geronoms (older people care professionals), and care assistants. The physiotherapists and social advisors in these facilities also participated in the immediate patient care of the older people and thus were included as care personnel. In Finland, nurse directors usually work as chief nurses or directors of nursing services, and nurse managers under them serve as immediate superiors to care personnel. Deputy nurse managers assist or substitute for nurse managers in managerial work, and especially deputy nurse managers participate in immediate patient care as well. Two data sets were collected simultaneously to explore factors and staff outcomes related to retention at multiple levels, which has previously been rarely studied.

Nursing staff in full‐ or part‐time positions during the data collection period were invited by their superiors via email to participate in this study. The invitation included information about the study and a link to the electronic survey. All units were also visited by a member of the research group, and during these visits, paper versions of the survey were left in the units. Prior to data collection, a power analysis was conducted using RAO Software (McDonald 2014). A population size of 97 nurse leaders and 333 care personnel was required to ensure a 5% margin of error and 95% confidence level. A total of 97 nurse leaders (response rate 76%) and 503 care personnel participated (response rate 21%) in the study.

### 3.3 Instruments

The Transformational Leadership Scale (TLS) was used to investigate perceptions of nurse leaders' practices among care personnel. It comprises 54 questions organized into five sub‐scales and scored on a 5‐point Likert scale (1 = strongly disagree, 5 = strongly agree). The subscales about nurse leaders include leadership ethics (14 questions), managing the nursing process (16 questions), feedback and rewards (6 questions), and professional development (7 questions). Nurse leaders responded to the questions on the nursing director sub‐scale, and care personnel responded to the questions on the nurse leader sub‐scales. Previous studies have reported Cronbach's alpha values from 0.90 to 0.97 for the subscales of this scale. (Kvist et al. 2019).

The Conditions for Work Effectiveness Questionnaire II (CWEQ‐II) measures structural empowerment and consists of 21 items measured on a 5‐point Likert scale. It has six sub‐scales: access to opportunity (3 questions), access to information (3 questions), access to support (3 questions), access to resources (3 questions), formal power (3 questions), informal power (4 questions), and global empowerment (2 questions) as a validation index. (Laschinger 2012). Previous studies have reported Cronbach's alpha values from 0.78 to 0.94 for this scale (Ta'an et al. 2020). In this study, this instrument was only used for nurse leaders. Additionally, care personnel were asked two questions concerning participation in the decision‐making about their work, and for career advancement in the organization, using 5‐step Likert scale.

### 3.4 Data Collection

Data collection took place between November 2022 and April 2023. The survey took approximately 20 min to complete. It included questions concerning socio‐demographic factors (*n* = 3) and setting characteristics (*n* = 6) questions as background factors as well as two previously developed instruments: the Transformational Leadership Scale (TLS) (Kvist et al. 2019) and the Conditions for Work Effectiveness Questionnaire II (CWEQ‐II) (Laschinger 2012).

The sociodemographic questions included age, gender, and education. As setting characteristics, participants were asked about their position, workplace, and work experience. Nurse leaders were also asked about the number of units, clients, and employees they were responsible for. As staff outcomes, all participants were asked to evaluate their work‐related well‐being, overall health, job satisfaction, valuing one's own work, commitment to the healthcare profession, and commitment to their current workplace on a visual analogue scale from 0 (weakest) to 10 (strongest).

### 3.5 Data Analysis

The data were analysed using SPSS 25.0 (IBM Corp.) for Windows. Demographic information was analysed using descriptive statistics. Participants were regrouped into approximately similar‐sized groups based on age, work experience in the social care and health sector, and, for leaders, the number of clients, units, and employees in their remit. Missing data were present for some variables. Analyses were conducted using pairwise deletion, whereby all available observations were included in each analysis, resulting in slight variation in sample size across analyses. (Field 2013.) Information on valid sample sizes is reported in the Results tables.

The sum variables of the instruments were calculated as per previous descriptions of their use (Kvist et al. 2019; Laschinger 2012). Spearman's correlation was chosen, because of the skewness in the data. It was used to examine the possible relationships between different variables: *ρ* values ≤ 0.30 indicate weak correlation and *ρ* values between 0.31 and 0.50 indicate moderate correlation. (Field 2013). The non‐normality of the data was tested with histograms and values of skewness and kurtosis, and the Kolmogorov–Smirnov test. The Mann–Whitney *U* test and the Kruskal‐Wallis test were used to investigate the possible differences between groups, such as age or education. In this study, *p* < 0.05 was considered statistically significant. Cronbach's alpha was used to examine the internal consistency of the sum variables, with values > 0.70 regarded as good, and values between 0.60 and 0.70 regarded as acceptable. Linear regression analysis was used to explain nursing staff members' commitment to their profession and workplace. Cook's distance was used in the regression analysis to find influential outliers in a set of predictor variables. The Cook's distance levels below one indicate small residuals and little leverage. Multicollinearity was assessed using variance inflation factors (VIF) and tolerance values. Residual normality was evaluated using Q–Q plots. Homoscedasticity was examined by inspecting residuals versus fitted values. (Field 2013).

### 3.6 Ethical Considerations

This study obtained research approval from the target organization as per their requirements. Based on Finnish legislation, it did not require ethical approval as the participants were nursing staff and the study did not concern patients or clients. In this study the participants were given the opportunity to ask questions before participating in the study. Participants gave their informed consent on the first page of the survey. Data were collected anonymously and stored such that only the designated members of the research group could access it. The two scales were used with the permission of their developers. (Finnish National Board on Research Integrity TENK 2021).

## 4 Results

### 4.1 Participants' Background Factors

The nurse leaders were mainly female (95%) and ranged in age from 25 to 66 years (mean 51 years). A majority (90%) of them were in permanent employment, mainly in managerial roles (81%). Half of this group had a master's or equivalent degree, 29% a bachelor's or equivalent degree, and 22% an upper secondary education. On average, the nurse leaders had 24 years (range 2–43 years) of experience in the healthcare sector and 10 years (range 0.5–34 years) in a leadership position. On average, nurse leaders had 33 employees (ranging from 1 to 270) and led 2 units (range 0–15). The number of clients in the units ranged from 0 to 272 (mean 41) (Table 1).

**TABLE 1:** Nurse leaders' demographics (*N* = 97).

Demographics	f	%
Gender (*n* = 97)		
Male	5	5.2
Female	92	94.8
Age in years (*n* = 97)		
25–47	35	36.1
48–57	29	29.9
58–66	33	34.0
Level of education (*n* = 97)		
Upper secondary education	21	21.6
Bachelor's or equivalent	28	28.9
Master's or equivalent	48	49.5
Type of employment (*n* = 97)		
Permanent employment	87	90.0
Temporary employment	8	8.0
Other	2	2.0
Managerial level (*n* = 96)		
Deputy nurse manager/charge nurse	8	8.2
Manager	78	80.4
Director	10	10.3
Number of employees (*n* = 94)		
0–20	31	33.0
21–29	31	33.0
30–270	32	34.0
Experience in healthcare in years (*n* = 95)		
1–15	23	24.2
16–29	37	38.9
30–43	35	36.8
Working in the current workplace in years (*n* = 94)		
0–2	40	42.6
3–10	27	28.7
11–34	27	28.7
Managerial experience in years (*n* = 76)		
0.5–5	35	46.1
6–10	14	18.4
11–15	22	28.9
16–34	5	6.6
Number of units (*n* = 93)		
0–1	40	43.0
2	31	33.3
3–15	22	23.7
Amount of clients in the units (*n* = 97)		
0–24	29	29.9
25–38	33	34.0
39–272	35	36.1

Most (86%) of the care personnel were female. Over half (64%) of them had an upper secondary education, and 25% a bachelor's or equivalent degree. Their mean age was 45 years (range 19–70 years). Their most common profession was practical nurse (60%), followed by registered nurse (20%), and 12% physiotherapist or social advisor. On average, they had 14 years (range 0–45 years) of experience in the healthcare sector and had been in their current workplace for 6 years (range 0–43 years). Most (79%) of them were employed permanently, on dayshifts (57%) or day‐ and nightshifts (37%) (Table 2).

**TABLE 2:** The care personnel's demographics (*N* = 503).

Demographics	f	%
Gender (*n* = 498)		
Male	40	7.6
Female	449	85.9
Other	1	0.2
Don't want to say	8	1.5
Age in years (*n* = 483)		
19–34	125	23.9
35–46	115	22.0
47–55	112	21.4
56–70	131	25.0
Level of education (*n* = 499)		
Student	5	1.0
Upper secondary education	332	63.5
Bachelor's or equivalent	131	25.0
Master's or equivalent	29	5.5
Profession (*n* = 499)		
Registered nurse	106	20.3
Practical nurse	316	60.4
Nurses' aide	12	2.3
Other	65	12.4
Type of employment (*n* = 495)		
Permanent employment	393	79.0
Temporary employment	69	14.0
Other	33	7.0
Experience in healthcare in years (*n* = 491)		
0–5	132	25.2
6–10	106	20.3
11–20	132	25.2
21–45	121	23.1
Working in the current workplace in years (*n* = 490)		
0–1	112	21.4
2–3	110	21.0
4–5	76	14.5
6–10	89	17.0
11–43	103	19.7
Form of working hours (*n* = 499)		
Day shift	298	57.0
Day and night shifts	193	36.9
Night shifts	5	1.0
Other	3	0.6

### 4.2 Evaluations of Staff Outcomes

The mean score for work‐related well‐being among nurse leaders was 7.9, overall health 8.5, and job satisfaction 7.7 (Table 3). A comparison between age groups showed that nurse leaders aged 58–66 (mean rank 58.85) had higher job satisfaction than those aged 25–47 years (mean rank 41.47, *p* = 0.028). The mean score for valuing one's own work was 8.8 (Table 3), and older nurse leaders (age group 58–66) reported higher levels of valuing their own work than younger ones (age‐group 25–47, mean rank 41.67, *p* = 0.031). Furthermore, nurse directors (mean rank 67.40) reported higher levels of valuing one's own work than other nurse leaders (mean rank 46.97, *p* = 0.045). The mean score for nurse leaders' commitment to the profession was 9.0 and to their current workplace 8.8 (Table 3). Nurse leaders' commitment to their current workplace was higher (mean rank 57.85) in the age group 58–66 than in the age group 25–47 (mean rank 38.30, *p* = 0.008). Nurse leaders with 16–29 years' work experience in healthcare reported higher commitment (mean rank 51.08) to the current workplace than those with 1–15 years' work experience (mean rank 36.24, *p* = 0.046).

**TABLE 3:** Nurse leaders' and care personnel's outcomes (*N* = 600).

Outcomes	Nurse leaders (*N* = 97)					Care personnel (*N* = 503)				
	*n*	Min	Max	Mean	SD	*n*	Min	Max	Mean	SD
Overall health	97	5	10	8.46	1.05	500	3	10	8.02	1.47
Work‐related well‐being	97	3	10	7.93	1.48	500	0	10	7.58	1.83
Job satisfaction	97	2	10	7.69	1.78	500	4	10	7.59	1.00
Valuing one's own work	97	2	10	8.80	1.32	502	0	10	8.69	1.60
Commitment to the profession	97	4	10	8.99	1.66	500	0	10	8.12	2.17
Commitment to the current workplace	97	2	10	8.76	1.41	501	0	10	8.40	1.87

The average score for care personnel's work‐related well‐being was 7.6, overall health 8.0, and job satisfaction 7.6. The mean score for valuing one's own work was 8.7, commitment to the profession 8.1, and commitment to their current workplace 8.3. The care personnel aged 56–70 were more committed to the profession (mean rank 285.24) than those aged 19–34 (mean rank 198.65, *p* < 0.001) or 47–55 (mean rank 233.96, *p* = 0.022) (Table 3).

### 4.3 Evaluations of Transformational Leadership Style and Structural Empowerment

Nurse leaders' average assessment of their superior's transformational leadership style was 3.91 (range 1–5, SD 0.87). They assessed opportunity (mean 4.1, SD 0.75) and information (mean 4.1, SD 0.68) as the strongest dimensions of structural empowerment, while resources (mean 3.0, SD 0.86) were the weakest dimension. Their mean score for empowerment overall was 21.3 (Table 4).

**TABLE 4:** Nurse leaders' structural empowerment (*N* = 97).

Dimension	No. of items	*n*	Min	Max	Mean	SD	α
Opportunity	3	96	2	5	4.11	0.75	0.79
Information	3	96	2	5	4.10	0.69	0.79
Support	3	97	1	5	3.26	0.82	0.77
Resources	3	95	1	5	2.99	0.86	0.77
Formal power (JAS)	3	96	2	5	3.27	0.77	0.77
Informal power (ORS)	4	96	2	5	3.66	0.72	0.77
GE	2	97	2	5	3.71	0.88	0.91
Total empowerment	21	91	13	29	21.31	3.18	0.91

*Note:* α = Cronbach's alpha.

The mean score for care personnel's assessment of their leaders' transformational leadership style was 4.0. The subscale leadership ethics received the most positive assessment (mean 4.17), while the subscale feedback and rewards were the weakest (mean 3.85) (Table 5). The mean score for care personnel's assessment of their opportunities to participate in decision‐making about their work was 2.6, and the mean score for their assessment of the opportunities for career advancement in the organization was 3.5.

**TABLE 5:** Care personnel's evaluation of their superior's transformational leadership style (*N* = 503).

Dimension	No. of items	*n*	Min	Max	Mean	SD	α
Leadership ethics	14	460	1	5	4.17	0.97	0.91
Managing nursing process	16	446	1	5	3.99	0.94	0.91
Feedback and rewards	6	468	1	5	3.85	1.02	0.91
Professional development	7	472	1	5	4.10	0.92	0.92

*Note:* α = Cronbach's alpha.

### 4.4 Associations Between Transformational Leadership, Structural Empowerment, and Staff Outcomes

Nurse leaders' assessment of their superior's transformational leadership style was found to correlate positively with their work‐related well‐being (*ρ* = 0.342, *p* < 0.01) and their job satisfaction (*ρ* = 0.437, *p* < 0.001). This indicates that the more transformational their superior's leadership style was, the stronger their own work‐related well‐being and job satisfaction were. Similarly, superiors' transformational leadership style correlated positively with nurse leaders valuing one's own work (*ρ* = 0.384, *p* < 0.001), commitment to their current workplace (*ρ* = 0.371, *p* < 0.001), and multiple dimensions of nurse leaders' structural empowerment (Table 6), particularly support (*ρ* = 0.545, *p* < 0.01).

**TABLE 6:** Correlations between nurse leaders' structural empowerment and associated factors.

	Nurse leaders' total structural empowerment		Information		Opportunity		Support		Resources		Formal power (JAS)		Informal power (ORS)	
	*ρ*	*p*	*ρ*	*p*	*ρ*	*p*	*ρ*	*p*	*ρ*	*p*	*ρ*	*p*	*ρ*	*p*
Superior's transformational leadership	0.540	< 0.01	0.295	< 0.01	0.177	0.086	0.545	< 0.01	0.372	< 0.01	0.419	< 0.01	0.412	< 0.01
Work‐related well‐being	0.390	< 0.001	0.237	< 0.05	0.115	0.264	0.303	< 0.01	0.410	< 0.01	0.370	< 0.001	0.305	< 0.01
Overall health	0.204	0.052	0.092	0.372	0.064	0.536	0.115	0.264	0.199	0.053	0.124	0.229	0.249	< 0.05
Job satisfaction	0.402	< 0.001	0.166	0.105	0.111	0.282	0.381	< 0.001	0.405	< 0.001	0.418	< 0.001	0.287	< 0.01
Valuing one's own work	0.316	< 0.001	0.204	< 0.05	0.263	< 0.01	0.186	0.068	0.118	0.255	0.305	< 0.01	0.195	0.057
Commitment to profession	0.378	< 0.01	0.320	< 0.01	0.184	0.074	0.320	< 0.01	0.202	0.050	0.190	0.063	0.201	< 0.05
Commitment to current workplace	0.355	< 0.01	0.161	0.118	0.268	< 0.01	0.272	< 0.01	0.263	< 0.05	0.290	< 0.01	0.255	< 0.05

Abbreviations: JAS, job activities scale; ORS, organizational relationships scale.

Another positive correlation was found between nurse leaders' work‐related well‐being and dimensions of their structural empowerment, particularly resources (*ρ* = 0.410, *p* < 0.01). Nurse leaders' job satisfaction also correlated positively with dimensions of their structural empowerment, most strongly with resources (*ρ* = 0.405, *p* < 0.001). The correlation between formal power and valuing one's own work (*ρ* = 0.305, *p* < 0.01) suggests that the more formal power nurse leaders have, the more they value one's own work. Further positive correlations (Table 6) were found between nurse leaders' overall structural empowerment and their commitment to the profession (*ρ* = 0.378, *p* < 0.01) and commitment to their current workplace (*ρ* = 0.355, *p* < 0.01). Care personnel's assessment of their leaders' transformational leadership style correlated positively with their job satisfaction (*ρ* = 0.388, *p* < 0.001).

### 4.5 Factors Explaining Staff's Commitment to Current Workplace and Profession

Based on the correlations found, the following variables were chosen as explanatory variables for the overall regression analysis of nurse leaders' commitment to the current workplace: valuing one's own work, commitment to the profession, work‐related well‐being, total structural empowerment, and superior's transformational leadership style. The statistically significant overall regression (R^2^ = 0.313, F (5, 84) = 9.124, *p* = < 0.001, Cook's distance 0.405) revealed that these factors explain 31% of nurse leaders' commitment to their current workplace. Once the statistically insignificant factors (valuing one's own work, structural empowerment, and superior's transformational leadership) were removed from the model, the final regression model suggested that nurse leaders' work‐related well‐being and commitment to the profession explained 37% of their commitment to their current workplace (R^2^ = 0.366, F (2, 94) = 28.660, *p* = < 0.001, Cook's distance 0.622). VIF (1.066) and tolerance values (0.938) indicated no multicollinearity. Furthermore, the residuals showed no substantial deviations from normality and suggested homoscedasticity (Table 7).

**TABLE 7:** Regression analysis of nurse leaders' commitment to current workplace or profession and other factors.

Commitment to current workplace				
Variables	Model 1		Final model	
	β	*p*	β	*p*
Commitment to profession	0.420	< 0.001	0.443	< 0.001
Work‐related well‐being	0.389	< 0.001	0.455	< 0.001
Valuing one's own work	0.044	0.711		
Superior's transformational leadership	0.295	0.156		
Structural empowerment	−0.037	0.529		
Model characteristics	*R* ^2^ = 0.313, F (5, 84) = 9.124, *p* = < 0.001, Cook's distance 0.405		*R* ^2^ = 0.366, F (2, 94) = 28.660, *p* = < 0.001, Cook's distance 0.622	

Commitment to profession		
	Model 1	
Variables	β	*p*
Valuing one's own work	−0.172	0.066
Commitment to current workplace	0.643	< 0.001
Superior's transformational leadership	−0.111	0.297
Work‐related well‐being	0.057	0.583
Structural empowerment	0.157	0.152
Model characteristics	*R* ^2^ = 0.404, F (5, 81) = 12.646, *p* = < 0.001, Cook's distance 0.682	

Similarly, the following factors were selected, based on the earlier correlations found, to explain care personnel's commitment to their current workplace: work‐related well‐being, valuing one's own work, commitment to the profession, opportunity to participate in decision‐making about one's own work, opportunities for career advancement in the organization, and nurse leader's transformational leadership. After filtering one outlier from the data, the overall regression was statistically significant (R^2^ = 0.425, F (6, 400) = 50.923, *p* = < 0.001, Cook's distance 0.209), and these factors together explained 43% of commitment to their current workplace among care personnel. Removing the statistically insignificant explanatory variables (the opportunity to participate in decision‐making and the opportunities for career advancement in the organization) from the analysis did not change the outcome in the final model (R^2^ = 0.431, F (4, 404) = 76.425, *p* = < 0.001, Cook's distance 0.282). VIF (1.149–1.473) and tolerance values (0.679–0.871) indicated no multicollinearity. Furthermore, the residuals showed no substantial deviations from normality and suggested homoscedasticity (Table 8).

**TABLE 8:** Regression analysis of care personnel's commitment to current workplace or profession and other factors.

Commitment to current workplace				
	Model 1		Final model	
Variables	β	*p*	β	*p*
Valuing one's own work	0.298	< 0.001	0.262	< 0.001
Commitment to profession	0.279	< 0.001	0.323	< 0.001
Nurse leader's transformational leadership	0.305	< 0.001	0.150	< 0.001
Work‐related well‐being	0.183	< 0.001	0.171	< 0.001
Possibility to participate on decision‐making regarding own work	−0.012	0.884		
Opportunities to advance in career	0.072	0.269		
Model characteristics	*R* ^2^ = 0.425, F (6, 400) = 50.923, *p* = < 0.001, Cook's distance 0.209		*R* ^2^ = 0.431, F (4, 404) = 76.425, *p* = < 0.001, Cook's distance 0.282	

Commitment to profession				
	Model 1		Final model	
Variables	β	*p*	β	*p*
Valuing one's own work	0.317	< 0.001	0.292	< 0.001
Commitment to current workplace	0.340	< 0.001	0.357	< 0.001
Work‐related well‐being	0.090	0.039	0.133	< 0.01
Nurse leader's transformational leadership	−0.048	0.279		
Possibility to participate on decision‐making regarding own work	−0.079	0.099		
Opportunities to advance in career	−0.086	0.056		
Model characteristics	*R* ^2^ = 0.41, F (6, 400) = 47.660, *p* = < 0.001, Cook's distance 0.280		*R* ^2^ = 0.403, F (3, 491) = 112.025, *p* = < 0.001, Cook's distance 0.344	

The variables for the overall regression model for care personnel's commitment to the profession were: work‐related well‐being, valuing one's own work, commitment to the current workplace, opportunity to participate in decision‐making about one's own work, opportunities for career advancement, and nurse leaders' transformational leadership. The overall regression was statistically significant (R^2^ = 0.41, F (6, 400) = 47.660, *p* = < 0.001, Cook's distance 0.280), with the selected factors together explaining 41% of commitment to the profession among care personnel. Removing from the analysis the statistically insignificant explanatory variables (opportunity to participate in decision‐making, opportunities for career advancement in the organization, and nurse leaders' transformational leadership) did not change the outcome in the final model (R^2^ = 0.403, F (3, 491) = 112.025, *p* = < 0.001, Cook's distance 0.344). VIF (1.212–1.537) and tolerance values (0.650–0.825) indicated no multicollinearity. Furthermore, the residuals showed no substantial deviations from normality and suggested homoscedasticity (Table 8).

## 5 Discussion

This study focuses on transformational leadership style, structural empowerment, and staff outcomes in nursing home settings. Previous studies on the topic have mainly been conducted in acute care settings (Cummings et al. 2018; Jarden et al. 2020; Niinihuhta and Häggman‐Laitila 2022) and have provided little consideration for nurse leaders (Kelly et al. 2019; Niinihuhta et al. 2022a) and their evaluations of structural empowerment (Laschinger 2012; Spencer and McLaren 2017).

The other novel insight offered by this study is that transformational leadership was assessed by two datasets at two different management levels at the same time and in the same organization. This enables us to consider how this leadership style, which is regarded as a key element in positive staff outcomes (Cummings et al. 2018; Marufu et al. 2021; Niinihuhta and Häggman‐Laitila 2022; Niinihuhta et al. 2022a; Hult et al. 2023), is implemented in an organizational culture. Our results showed that both levels of the organization demonstrated a good level of transformational leadership. This could be seen as a significant resource for the organization because of the positive associations with work‐related well‐being (Cummings et al. 2018; Hult et al. 2023; Niinihuhta and Häggman‐Laitila 2022; Niinihuhta et al. 2022a) and with retaining nursing staff (Cummings et al. 2018; Hult et al. 2023; Marufu et al. 2021). Interestingly, our study did not find a correlation between nurse directors' leadership style and outcomes for care personnel. This finding may be associated with the fact that care personnel assessed their immediate superiors (nurse managers) as did the nurse managers (nurse directors). Furthermore, the number of nurse directors was substantially lower than nurse managers in the organization, and they do not usually have direct contact with the care personnel. Despite the lack of direct contact with the care personnel, nurse directors present a role model for the nurse managers, which may positively guide their leadership style on the care personnel. However, exploring this association would require further research.

Nurse leaders, particularly older ones, assessed their work‐related well‐being, overall health, and job satisfaction more highly than care personnel did, although their assessments were also positive. Nurse leaders also reported higher commitment to both the profession and their current workplace than care personnel did. The higher the nurse leader's managerial level, the higher they valued their own work. These results concerning nurse leaders align with previous studies (Kelly et al. 2019; Niinihuhta et al. 2022b) which have shown that nurse leaders have ways of coping with their demanding work (Labrague et al. 2018). Similarly, older care personnel were more committed to the profession than younger personnel. These results could reflect differences between generations in nursing (Cziraki et al. 2020) and should be kept in mind during the development of healthy work environments and further studies. It is worth noting that the “survivor effect” in older professionals could be associated with higher work‐related well‐being and job satisfaction, meaning that those who liked their work and workplace were more likely to be in the study sample than those who didn't and left.

Based on our results, older nurse leaders and care personnel represent a resilient employee group and, as such, a remarkable resource for the organization. This will be reduced over the coming years as these staff retire. Organizations may therefore consider ways to improve commitment among younger personnel and ensure their career progression through long‐term employment relationships. One approach could be to enhance provision of those aspects that nurses appreciate in their working environment (Mabona et al. 2022; Moloney et al. 2020). Individualized mentoring programs that address core and specific competencies have been associated with decreasing nurse turnover, particularly among young personnel (Vázquez‐Calatayud and Eseverri‐Azcoiti 2023). However, expectations about good work environments are dynamic, and more research is needed on this topic.

Nurse leaders and care personnel assessed their superior's transformational leadership more positively than in a previous Finnish study (Kvist et al. 2019). The weakest subscale was feedback and rewards, which aligns with that previous study (Kvist et al. 2019). Organizations and nurse leaders should focus more on improving mechanisms for giving feedback and rewards. Nurse leaders' ability to give positive feedback and rewards helps nursing staff to succeed and develop in their profession. It may also foster a sense of engagement with the organization and enhance nurses' work‐related well‐being (Holland et al. 2019; Moloney et al. 2020). Thus, it should be part of everyday leadership (Kvist et al. 2019). However, it has proven difficult to implement feedback and reward mechanisms, possibly because nurse leaders lack knowledge about methods that are effective and appreciated by their staff. Targeted education and intervention studies could contribute to improving these practices.

Nurse leaders assessed their structural empowerment as moderate in this study, which mirrors the findings of previous studies (e.g., Niinihuhta et al. 2022a; Spencer and McLaren 2017), indicating that there is still room for improvement. Nurse leaders' structural empowerment could be enhanced through opportunities for education, regular feedback, and peer support. These would further enhance the nurse leaders' resources in the form of increased knowledge and professional autonomy (Jafari et al. 2021; Marufu et al. 2021). This is noteworthy because our results indicate that resources, nurse leaders' work‐related well‐being, and job satisfaction are positively associated and may reinforce each other. Nurse leaders need to be supported in their structural empowerment as a whole, in order to have enough strength to make decisions that improve the staff's competencies, care for patients, and positive work environments for their staff. Our results also pointed out that the more formal power nurse leaders have, the more they value their own work. This should be taken into consideration by organizations as they develop the job descriptions and positions for nurse leaders, ensuring that they have sufficient authority and opportunities to use their expertise and lead their employees well.

As nurse leaders set an example for their employees, increasing their autonomy and participation in decision‐making in organizations could also encourage other personnel to participate more, an area that was evaluated rather negatively in the current study. This study also showed that care personnel lack career opportunities, echoing the findings of Cziraki et al. (2020). Mechanisms for improving this may include enhancing the structures for organizational participation (Cziraki et al. 2020; Moloney et al. 2020) and improving leaders' knowledge management (Cziraki et al. 2020).

The better care personnel assessed their leaders' transformational leadership style, the better their own job satisfaction was. Nurse leaders' supportiveness, civility, and interest in their employees' work‐related well‐being have been reported to be positively associated with nursing staff retention (Moloney et al. 2020). Strengthening relational leadership behaviours by educating nurse leaders and improving their collaboration skills, such as the ability to engage employees in decision‐making, should be a priority for policy makers and hospital administrators (Cziraki et al. 2020).

Our results of nurse leaders' and care personnel's commitment to their current position highlight the significance of work‐related well‐being. Our results concerning transformational leadership demonstrate that it is a valuable means for enhancing employees' work‐related well‐being and nurse leaders' structural empowerment. As such, it appears to be an important consideration in efforts to support staff retention.

### 5.1 Strengths and Limitations of the Work

The strengths of the study are prior to data collection conducted power analysis, the good response rate among nurse leaders (76%), and relatively low response rate among care personnel (21%), which is however comparable to some recent nursing studies (Timmins et al. 2023). Lower response rate among care personnel can cause non‐response bias and thus offer an inadequate representation of the population (Sammut et al. 2021). We did not analyse the failure in the response rates and reasons behind them, and this can be regarded one limitation of our study. Reasons for low participation of health care professionals can be for example uninterest in research, high workload, time restrictions and survey fatigue (Sammut et al. 2021; Shiyab et al. 2023; Timmins et al. 2023). Based on previous studies, several reminders (Sammut et al. 2021; Shiyab et al. 2023; Timmins et al. 2023), closer personal contact to researchers (Shiyab et al. 2023) and the traditional survey method with paper format could provide higher response rate compared to web survey (Sammut et al. 2021). We used all these, but they did not improve our response rate.

Concerning further our data collection, the use of internationally established instruments with good internal consistency (α ≥ 0.70) further strengthens this study. In addition, a statistician was consulted. It should be noted that commitment to the workplace and commitment to the profession may be difficult to interpret in a cross‐sectional design due to the possible circularity. Although in this study the diagnostics (VIF, tolerance, Q–Q plot, and residuals‐versus‐fitted plot) showed no multicollinearity and the regression assumptions were met (Field 2013), the potential circularity between these constructs should be taken into account in future research.

This study was conducted in Finland in one large organization, and this could be seen as a limitation, although this organization provides services to over 630,000 of Finland's 5.5 million inhabitants. The fact that the participants were not linked at individual or unit level to each other may be seen as a further limitation that should be addressed in future studies. In addition, the data were collected using a self‐reported survey, which should be taken into account in the interpretation of the results. Measuring the structural empowerment of the care personnel using the same instrument as used for nurse leaders could have strengthened the study and enhanced comparability. This should be acknowledged in future research. Most nurse leaders who participated were nurse managers, and most care personnel participants were practical nurses, which must be kept in mind when considering the representation of the nursing staff in nursing home settings.

### 5.2 Implications for Practice

The study population from a large organization and from its various units represents well Finnish nursing homes providing both long‐term and short‐term care, as well as their personnel and role responsibilities. Results are therefore conceptually and contextually transferable in Finland. The results of the current study provide nurse leaders with additional insight into aspects that warrant particular attention when leading complex nursing work environments and planning their own development. According to our results, besides having good capabilities in transformational leadership, nurse leaders may benefit from understanding how staff members' opportunities for decision‐making and professional development are associated with leadership practices. Our results suggest that capabilities related to providing rewards and feedback are relevant factors and should therefore be considered in nurse leaders' continuous training. Nurse leaders and care personnel may benefit from discussing and agreeing on joint practices for feedback and rewards. Organizations for their part have the possibility to enhance the visibility of feedback and encourage nurse leaders to integrate constructive feedback into the staff's regular activities. Furthermore, the given feedback should be regularly monitored and openly discussed. This is likely to be facilitated by a safe and trustworthy organizational culture. Nurse leaders should also concentrate on the means for enhancing work‐related well‐being for personnel, as work‐related well‐being has been associated with higher commitment to the workplace and perceived productivity, thereby contributing to organizational resources and social capital (Patrician et al. 2022). One approach to developing these means could be collaborating with occupational health and human resource units.

Nurse leaders and their superiors also need support from their organizations in terms of how to lead different generations with different needs. Older nursing staff are a remarkable resource for their organizations and their younger colleagues, and it is therefore worth investing in them. Nurse leaders should also find ways to promote peer support and mentoring opportunities between different age groups. On the nurse leaders' part, our results underline the need to improve their overall structural empowerment and opportunities to exercise formal power. This is a message for the nurse leaders' superiors. Nurse leaders themselves probably know best how this could be improved. Progress may be facilitated through mutual negotiations between nurse leaders and their superiors. The fact that nurse leaders' superior's leadership style did not correlate positively with care personnel outcomes raises a further area for development. This finding may suggest that superiors should pay more attention to the human resources in their units, to the challenges that the workforce faces in patient care and increase their support for nurse leaders in this respect.

## 6 Conclusion

Transformational leadership and structural empowerment appear to be important factors associated with positive staff outcomes in nursing home settings. Thus, these aspects may warrant consideration and further development within organizations, as they could be linked to efforts to address nursing shortages and support the quality of care provided. In addition, enabling staff participation in decision‐making related to their own work and offering career advancement opportunities may be beneficial, as these were observed at relatively modest levels. Nurse leaders' structural empowerment was also found to be at a moderate level in the current study, suggesting potential room for improvement within organizations. Attention to nurse leaders' access to resources and support may be particularly relevant, as these factors could be associated with their organizational commitment. Nursing homes are complex and currently understudied environments, highlighting the need for further research to explore different associations and potential explanatory factors in this context.

## Author Contributions

Conceptualization: Milja Niinihuhta, Anja Terkamo‐Moisio, Tarja Kvist, and Arja Häggman‐Laitila. Data curation: Milja Niinihuhta. Formal analysis: Milja Niinihuhta. Funding acquisition: Arja Häggman‐Laitila. Investigation: Milja Niinihuhta. Methodology: Milja Niinihuhta, Anja Terkamo‐Moisio, and Arja Häggman‐Laitila. Validation: Milja Niinihuhta. Supervision: Anja Terkamo‐Moisio, Tarja Kvist, and Arja Häggman‐Laitila. writing – original draft: Milja Niinihuhta. writing – review and editing: Milja Niinihuhta, Anja Terkamo‐Moisio, and Arja Häggman‐Laitila.

## Funding

VTR/ERVA, Grant/Award Number: HEL 2022‐006042 T 13 00 02, City of Helsinki, Social Services, Health Care and Rescue Services Division.

## Ethics Statement

The authors have nothing to report.

## Conflicts of Interest

The authors declare no conflicts of interest.

## Data Availability

The data that support the findings of this study are available from the corresponding author upon reasonable request.
